# Targeted Medical Consultation and Lifestyle Modification Advice for the Improvement of Quality of Life and Daily Habits in Gynaecological Patients: A Pilot Study

**DOI:** 10.7759/cureus.72015

**Published:** 2024-10-21

**Authors:** Kanelina Bimpa, Emmanouil M Xydias, Despoina Karagiannidou, Agapi Sagali, Elias Tsakos

**Affiliations:** 1 Breast Care, EmbryoClinic IVF, Thessaloniki, GRC; 2 Department of Obstetrics and Gynaecology, EmbryoClinic IVF, Thessaloniki, GRC

**Keywords:** diet, gynaecological patient, heath-related quality of life, lifestyle modification (lsm), physical activity, questionnaire survey

## Abstract

Introduction

Lifestyle factors account for a considerable part of the etiology of several metabolic disorders and many chronic benign and malignant conditions in the gynecological patient in particular. Lifestyle modification interventions offered within the context of gynecological care and endorsed by a medical practitioner have the potential to more strongly motivate patients to enact long-lasting changes.

Methods

A randomized pilot study was performed on 60 gynecological patients, utilizing simple lifestyle information and advice, supplementing routine consultation. Quality of life, dietary, exercise and smoking related habits were assessed at enrolment and then at 15, 30, and 60 days into the study.

Results

Quality of life significantly improved for the study group at 15- and 30-day assessments, but the difference was not maintained at 60 days. Participants in the study group significantly increased weekly fruit portions, but no differences were evident for vegetable, red meat, and dessert portions. Weekly exercise rate and exercise duration were higher in the study group, as was the adoption of a more physically active routine for non-exercising participants. Finally, the study group smokers managed to significantly reduce the number of daily cigarettes, but this was a small subgroup of the overall participants.

Conclusion

The findings of the present study indicate the potential of lifestyle interventions endorsed by a trusted medical practitioner for improving quality of life and daily habits in the gynecological patient. A larger, follow-up study is necessary to confirm these findings and further refine the interventions used, in order to maximize effectiveness.

## Introduction

The term “lifestyle” encompasses all daily behaviors, functions, and habits of an individual with regard to their diet, occupation, and recreation [1]. Lifestyle has historically been intrinsically linked to physical and mental health, a connection that is only reinforced as time passes, with the WHO reporting that over 60% of factors proven to exert an effect on an individual’s health and quality of life demonstrate a quantifiable correlation to lifestyle [2]. In particular, several conditions encountered in the context of female health have an etiologic connection with lifestyle factors. Apart from the well-known lifestyle-related disorders, such as obesity, type 2 diabetes mellitus (T2DM), liver disease, and metabolic syndrome; cancer risk may also be affected. With gynecological (uterine, cervical, and ovarian) and breast cancer accounting for over 35% of all malignancies occurring in women and for over 23% of malignancy-related mortality [3], lifestyle modification advice should constitute an indispensable part of routine clinical practice of female health experts and practitioners, as a preventive measure. 

While it could be argued that the promotion of a healthy lifestyle is widely practiced in the modern world, the actual data paint a bleaker picture. Overweight and obesity rates are on the rise globally, with female obesity and severe obesity (BMI > 40 kg/m^2^) being twice and thrice as prevalent respectively in 2017 compared to 1988 [4]. Physical inactivity and sedentary lifestyle are also becoming more and more prevalent globally [5] and smoking, while decreasing considerably in prevalence for women over the years, is still a major health hazard that affects millions worldwide [6]. Furthermore, the general public seems to be largely unaware of the true impact of lifestyle factors in the development of cancer, with physical inactivity in particular being considerably underestimated [7].

This lack of awareness and motivation could be addressed and resolved, in part, by healthcare professionals. Particularly, with regard to female healthcare, their expert-level knowledge on the impact of lifestyle factors on quality of life, chronic metabolic disorders, and gynecological malignancy risk in particular, combined with the generally high levels of trust (over 80%) medical practitioners enjoy from the public [8], could be utilized to promote lifestyle modifications and reduce cancer risk. The present pilot study aimed to test this hypothesis, via assessing the effect of simple health tips endorsed by a trusted medical professional on measurable lifestyle modifications and patient quality of life.

## Materials and methods

Study design

In order to assess the effectiveness of simple lifestyle modification advice offered by the physician, a pilot study employing a randomized, controlled design based on questionnaire was utilized. The questionnaire that constituted the basis of the study was the McGill Quality of Life Questionnaire - Expanded (MQLQ-E) [9], which offers a brief but thorough assessment of patient quality of life in multiple different domains that expand beyond just the physical symptoms experienced by the patients. The questionnaire was supplemented with additional questions regarding specific dieting, smoking, and exercising habits and how they were impacted by the advice received. Questionnaires were digitally distributed, and data were anonymously collected, stored in a de-identified manner, and interpreted without knowledge of the patients’ identity. The study protocol was reviewed and approved by EmbryoClinic's Scientific Board (Protocol Number: EC-2023003) on the 21st of April 2023. All participants provided informed written consent for their participation to the study.

Intervention

The present study aimed to assess the effectiveness of lifestyle modification advice coming from a figure of authority, namely, the patient’s own attending physician. In addition, the effectiveness of an automated system, with reduced burden on the physician and clinic themselves, was also tested. Therefore, for the present study, a collection of lifestyle modification tips was prepared by the clinic's medical team, worded in a patient-friendly manner focusing primarily on dieting, body weight loss, and exercise. Examples of such tips are the following: “How about skipping fried foods today?”, “You look beautiful in any attire. How about putting on your running shoes today?”, “Show your brightest smile to the world by cutting down on smoking”, "Strawberries are great at this time of year, how about trying some, instead of dessert?, "What a great day to take a walk by the beach!" etc. Bright, warm colors, a welcoming font, humorous language, wordplay, and positive images were used to create motivational cards with this lifestyle advice. Each card was accompanied by a “motivational coach” in the form of a friendly female figure of healthy bodily proportions, thus creating a friendly, non-intimidating companion for the patients, with an attainable physical condition. These cards were sent via a clinic-affiliated email to participants of the intervention group. These “friendlier” pieces of advice aimed at supplementing the more conventional patient-doctor consultation session that took place on the recruitment day and to attempt to maintain patient motivation and positive outlook with as low a workload burden for the clinic as reasonably achievable.

For the intervention (study) group, lifestyle advice was sent daily during the first 15 days of participation, every other day for the following 15 days and three times a week for the next month. This was done in order to ascertain the optimal balance of effectiveness and workload burden for the clinic. Specific advice from a pool of prepared cards was selected that corresponded to the needs of each patient (focus on dieting, weight loss, and/or exercise). For patients that actively smoked during recruitment, additional advice on cutting down on it was also sent, in the form of a separate email system. The control group received no email notifications.

Setting and participants

The study was conducted prospectively in a single, outpatient gynecology and breast health centre in Thessaloniki, Greece, and in total lasted from May 2023 to October 2023. Participants were patients attending the centre for routine gynecological and/or breast assessment screening or follow-up assessment after treatment of suspected or confirmed malignancy. Participants were randomly allocated into two groups using block randomization [10]. Patients were recruited during their visit to the clinic, where they completed a baseline questionnaire assessment (day 0). Subsequently, following routine physical examination, all patients were thoroughly consulted with regard to the need for lifestyle modification and the potential risks of not doing so for gynecological conditions. This was particularly stressed for patients with a positive prior history of intervention for suspected or confirmed malignancy. Finally, all participants completed follow-up questionnaires at 15, 30, and 60 days after their enrollment.

Questionnaire

Effectiveness of lifestyle modification advice was assessed with the McGill Quality of Life Questionnaire - Expanded (MQLQ-E) [9]. The MQLQ-E assesses quality of life using structured questions evaluating quality of life on a 10-point Likert scale [11], with possible values from 0 to 10. The 20 questions included in MQLQ-E are organized into eight distinct domains, each focusing on different aspects of quality of life, namely, D1: Physical (three questions) , D2: Psychological (four questions), D3: Existential (four questions), D4: Social (three questions), D5: Environment-safety of surroundings (one question), D6: Cognition - cognitive functions (two questions), D7: quality of health care (two questions), and D8: sense of becoming a burden to family and friends (one question). For domains with multiple questions, the average score of all domain questions was calculated, reflecting the performance of the domain as a whole. Both the overall score of the MQLQ-E questionnaire and the score of each individual domain were assessed and compared between the groups. The questionnaire was translated into Greek via back-translation by two independent translators, the resulting versions were synthesized into one, reviewed by a medical specialist and linguist and tested on a small contingent of the clinic’s patients to verify consistency. Supplementary questions regarding measurable impact and alteration of lifestyle and habits were included. These questions assessed body weight change, exercise habits, diet, and smoking habits.

The questionnaire was completed in digital form on the Google Forms platform (Google LLC, Mountain View, California, United States). Following consent to participation, participants were provided with a unique six-digit code, which acted as their identifier for the duration of the study. No identifying information (name, email address, etc.) was requested within the questionnaire form. Two investigators were responsible for participant enrollment and were the only ones with knowledge of patient name and study identifier correspondence. Two different investigators performed all data analyses and were blinded to participant identifying information.

Statistical analysis

Descriptive statistics were used for data presentation. Continuous variables were presented using mean, standard deviation, median, interquartile range, and minimum-maximum range. Categorical variables were presented as crude numbers and percentages. Questionnaire scores were compared between the intervention and control groups at every time-point of assessment using the two sample t-test or the Mann-Whitney test for continuous and the chi-square test for categorical variables. A p-value lower than 0.05 was considered statistically significant. All analyses were conducted using the IBM SPSS Statistics for Windows, Version 26.0 (IBM Corp., Armonk, NY).

## Results

**Figure 1:**
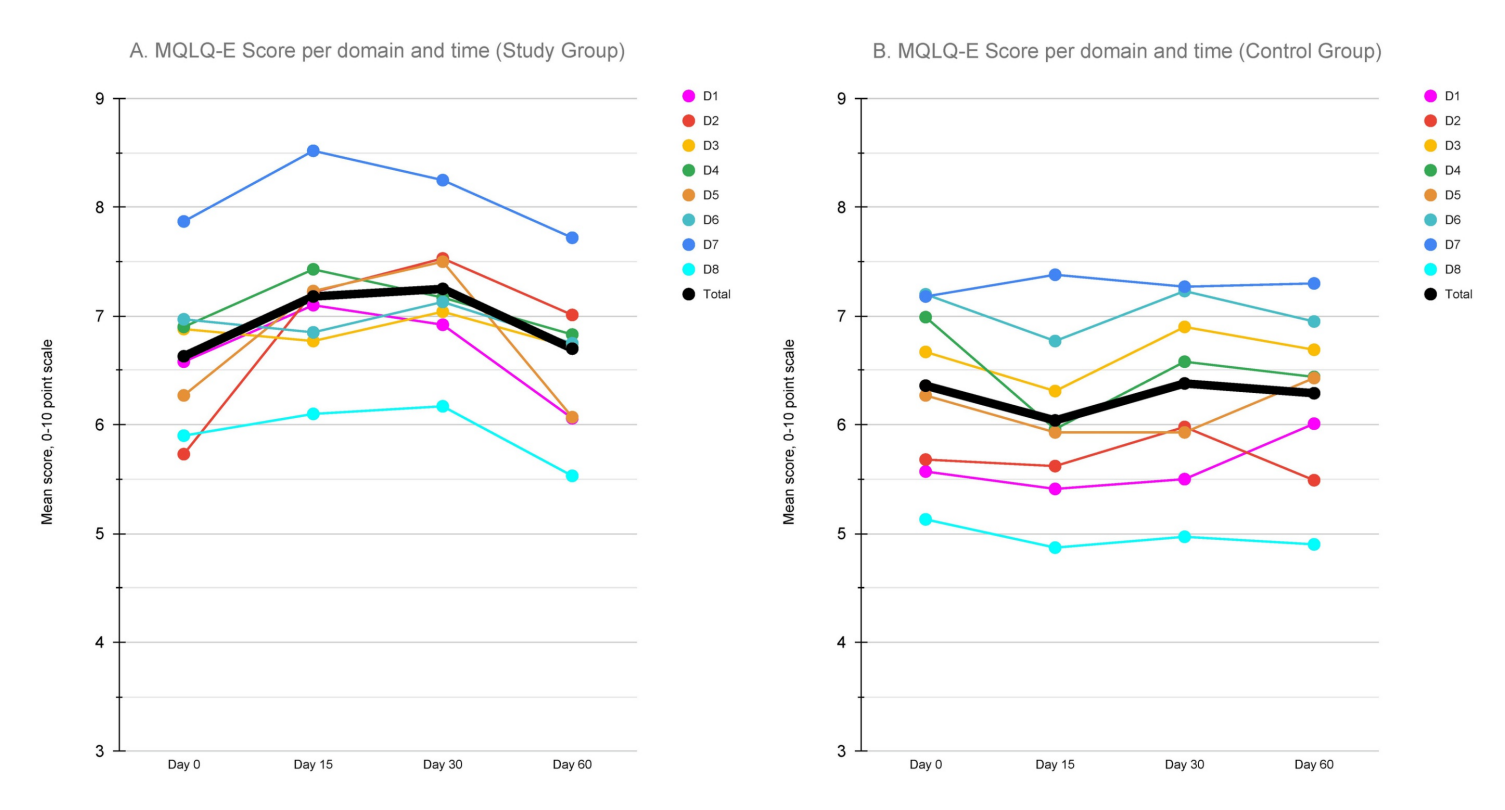
Line plot depicting the dynamic changes in MQLQ-E scores (per domain and overall) through the duration of the study. A: MQLQ-E scores in the study group, B: MQLQ-E scores in the control group. D1: Physical, D2: Psychological, D3: Existential, D4: Social, D5: Environment, D6: Cognition, D7: Healthcare, D8: Burden. MQLQ-E: McGill Quality of Life Questionnaire - Expanded

In total, 60 patients were recruited and were randomly allocated into two groups. Notable baseline participant characteristics were comparable between the intervention and control groups (Table 1). All participants completed all four study questionnaires. Regarding the effectiveness of the study intervention, the impact on overall MQLQ-E score and individual domain score are summarized on Table 2. Baseline assessment indicated no significant differences between the study and control groups. However, evaluation after 15 days indicated a significant overall quality of life improvement in the study group, with significant differences observed specifically in the physical, psychological, social, and healthcare domains. Significant difference in the overall score was maintained at the 30-day assessment, with significant differences in the same domains, except for social and healthcare, but with the addition of the environment domain. Finally, scores were not significantly different at 60 days, but the psychological domain remained significantly different between the study and control groups. Overall questionnaire scores for the entire duration of the study were significantly higher in the study group. The changes in overall and individual MQLQ-E domain scores throughout the study duration are graphically depicted on Figure 1.

**Table 1:** Baseline characteristics of the participants in the intervention and the control group. BMI: body mass index, SD: standard deviation, IQR: interquartile range

Parameter	Intervention (n = 30)	Control (n = 30)	P-value
Age, mean±SD, median (IQR, min-max)	50.6±9.64, 51 (12.9, 31.8-66.4)	46.6±9.10, 46.1 (13.9, 25.6-64.3)	0.104
BMI, mean±SD, median (IQR, min-max)	27.6±5.01, 27.1 (19-42)	26.2±5.44, 25.9 (17.6-43)	0.322
Breast clinic patients, n (%)	19 (63.4%)	13 (43.4%)	0.121
Prior history, n (%)	11 (36.6%)	17 (56.6%)	0.121
Smoking, n (%)	9 (30%)	6 (20%)	0.371

**Table 2:** Summary of the results of the McGill Quality of Life Questionnaire – Expanded (MQLQ-E) for the intervention and control groups throughout the study’s duration. D1: Physical, D2: Psychological, D3: Existential, D4: Social, D5: Environment, D6: Cognition, D7: health care, D8: burden, *: statistically significant difference (p < 0.05)

MQLQ-E domains per period		Intervention group				Control group				P-value
		Mean	SD	Median	Range	Mean	SD	Median	Range	
Baseline (day 0)	D1	6.58	2.22	6.50	2.67-10.0	5.57	2.07	5.00	1.33-10.0	0.073
	D2	5.73	1.67	5.75	2.75-8.25	5.68	1.80	5.50	3.25-8.75	0.999
	D3	6.88	1.60	6.50	3.00-10.0	6.67	1.81	6.75	3.00-9.75	0.638
	D4	6.90	1.99	7.33	2.33-9.67	6.99	2.07	6.33	3.67-10.0	0.959
	D5	6.27	2.50	7.00	0-9.00	6.27	2.63	6.00	0-10.0	0.905
	D6	6.97	2.18	7.50	2.00-10.0	7.20	1.88	7.00	2.50-10.0	0.658
	D7	7.87	1.75	7.50	5.00-10.0	7.18	1.87	7.00	3.50-10.0	0.150
	D8	5.90	3.17	6.50	0-10.0	5.13	3.17	5.00	0-10	0.353
	Total	6.63	1.40	6.60	3.90-8.95	6.36	1.34	6.05	4.55-8.80	0.391
1st assessment (day 15)	D1	7.10	1.56	7.00	4.00-10.0	5.41	1.69	5.33	1.67-9.33	<0.001*
	D2	7.22	1.46	7.38	4.00-10.0	5.62	1.72	5.50	3.00-8.75	<0.001*
	D3	6.77	1.59	6.63	4.00-9.50	6.31	1.69	6.13	4.00-10.0	0.190
	D4	7.43	1.51	7.67	4.33-10.0	5.96	1.50	5.67	3.00-9.00	<0.001*
	D5	7.23	2.30	8.00	2.00-10.0	5.93	2.68	5.00	0-10	0.070
	D6	6.85	1.71	7.00	2.50-9.00	6.77	2.08	6.25	1.50-10.0	0.866
	D7	8.52	1.85	9.00	3.00-10.0	7.38	1.58	7.50	3.50-10.0	0.004*
	D8	6.10	3.14	7.00	0-10.0	4.87	2.90	6.00	0-9.00	0.061
	Total	7.18	1.13	7.17	5.40-8.95	6.04	1.18	5.75	4.35-8.75	<0.001*
2nd assessment (day 30)	D1	6.92	1.78	7.00	3.33-10.0	5.50	1.92	5.67	1.00-9.33	0.004*
	D2	7.53	1.86	7.75	2.75-10.0	5.98	1.98	6.00	1.50-9.50	0.003*
	D3	7.04	1.51	7.00	2.75-9.50	6.90	1.98	7.00	1.00-10.0	0.756
	D4	7.17	1.60	7.33	4.67-10.0	6.58	2.19	6.33	1.00-10.0	0.239
	D5	7.50	1.68	8.00	4.00-10.0	5.93	2.68	6.00	0-10.0	0.009*
	D6	7.13	1.84	7.25	3.00-10.0	7.23	2.04	7.50	1.00-10.0	0.761
	D7	8.25	1.32	8.50	5.00-10.0	7.27	2.32	7.50	0-10.0	0.072
	D8	6.17	2.36	7.00	0-10	4.97	3.36	6.50	0-9.00	0.285
	Total	7.25	1.18	6.90	4.95-9.50	6.38	1.41	6.38	2.05-9.45	0.012*
3rd assessment (day 60)	D1	6.06	2.41	5.17	0.67-10.0	6.01	2.53	5.67	1.00-10.0	0.929
	D2	7.01	1.97	7.13	2.25-10.0	5.49	2.37	5.50	1.25-9.75	0.009*
	D3	6.71	1.81	6.50	3.25-9.25	6.69	1.96	6.75	2.25-10.0	0.973
	D4	6.83	1.67	6.67	4.00-10.0	6.44	2.10	5.50	3.00-10.0	0.351
	D5	6.07	2.61	7.00	0-9.00	6.43	2.53	6.00	1.00-10.0	0.737
	D6	6.75	1.84	7.50	2.50-10.0	6.95	2.24	7.00	2.00-10.0	0.638
	D7	7.72	1.74	8.00	5.00-10.0	7.30	2.34	7.50	0-10.0	0.436
	D8	5.53	2.64	6.00	0-9.00	4.90	3.35	5.00	0-10.0	0.517
	Total	6.70	1.46	6.53	3.90-9.40	6.29	1.61	6.38	2.45-9.20	0.298
Overall		6.94	1.09	6.87	5.42-8.96	6.27	1.19	6.21	3.39-8.70	0.026*

**Figure 2:**
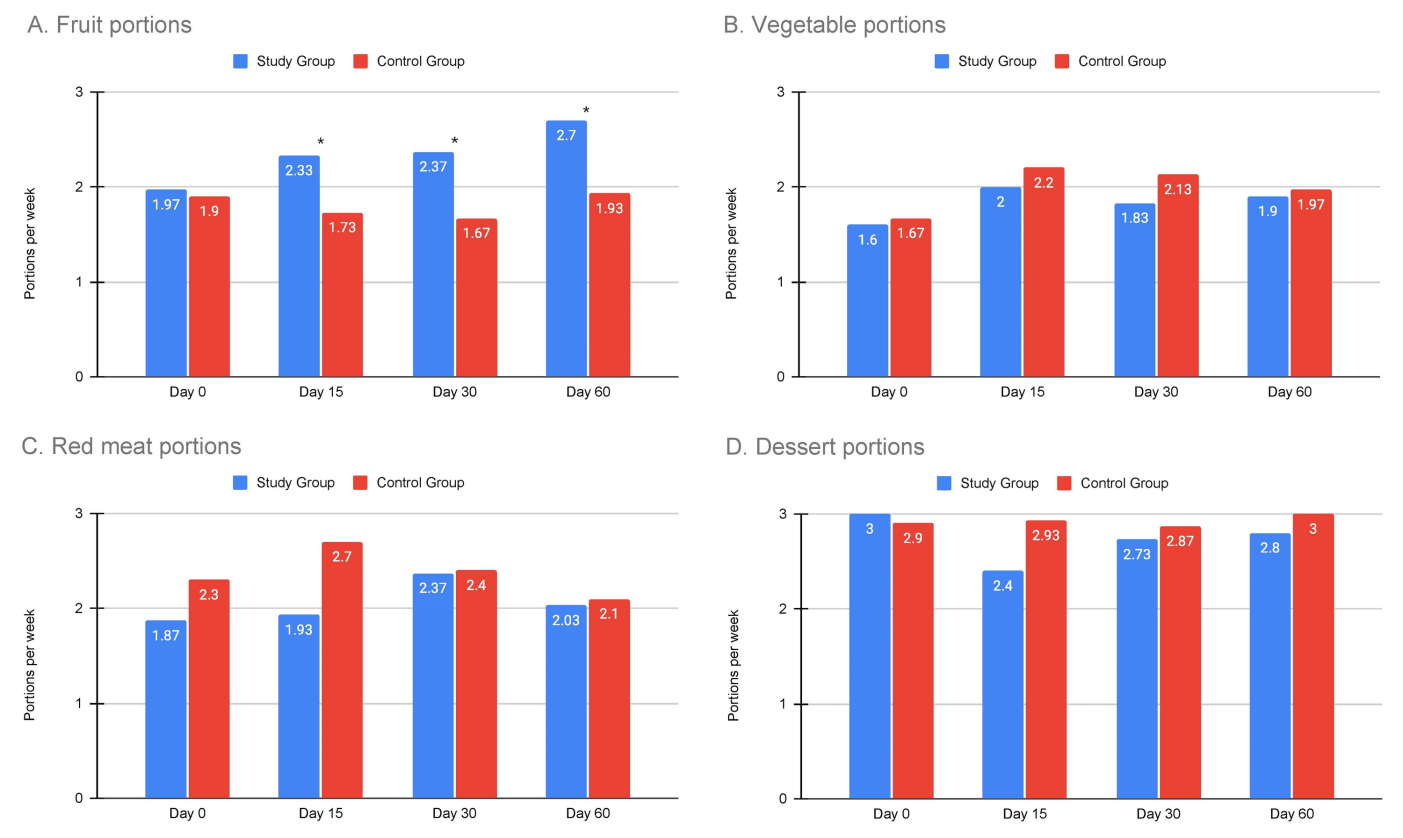
Bar charts depicting the changes in the participants’ weekly food portions for the four categories assessed. A: chart for fruit portions, B: chart for vegetable portions, C: chart for red meat portions, D: chart for dessert portions, *: statistically significant difference between study and control group (p < 0.05).

**Figure 3:**
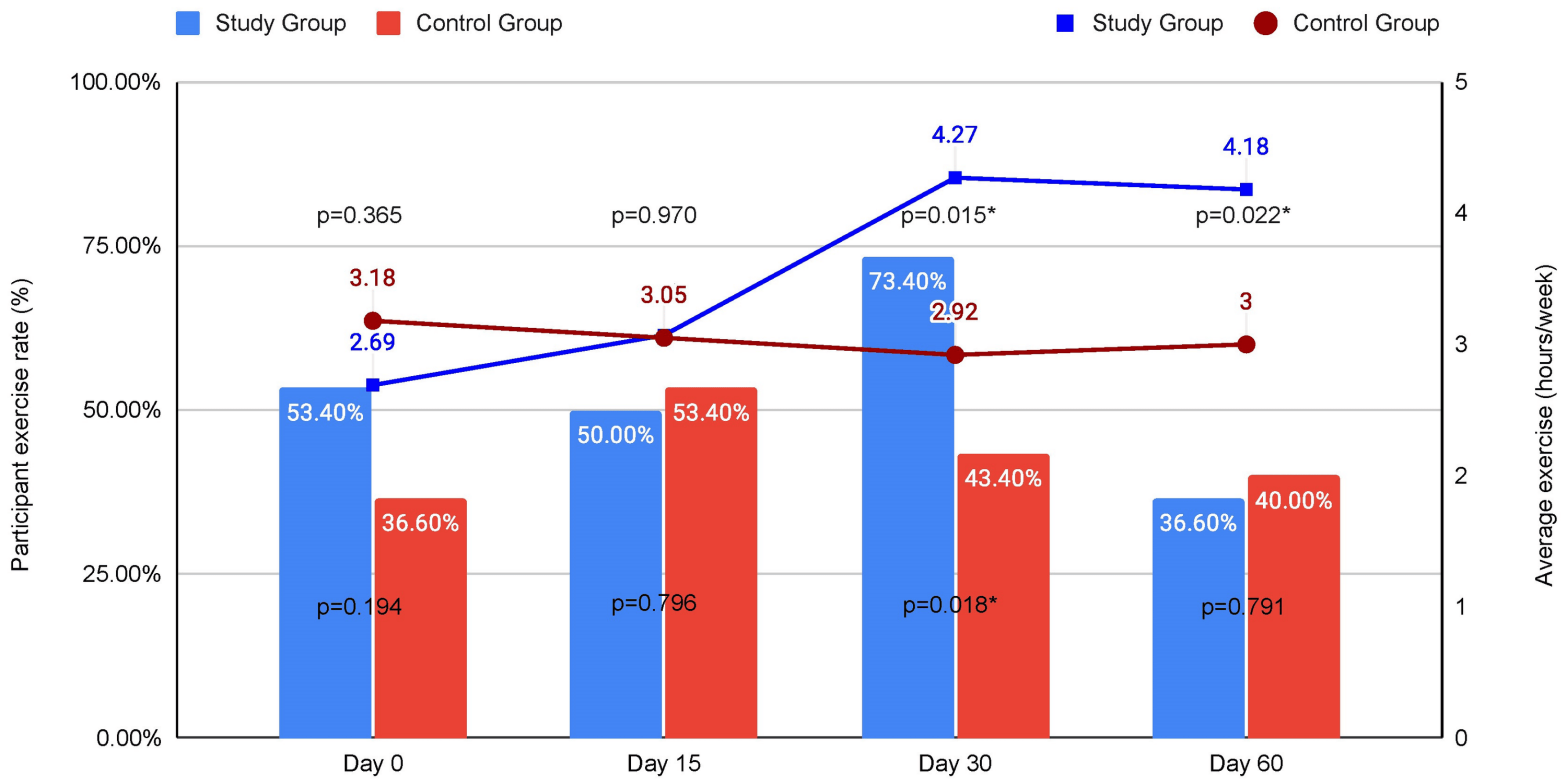
Combined bar and line chart depicting the percentage of participants who exercised weekly (bars) and their average exercise duration in hours every week (line). *: statistically significant difference between study and control group (p < 0.05).

**Figure 4:**
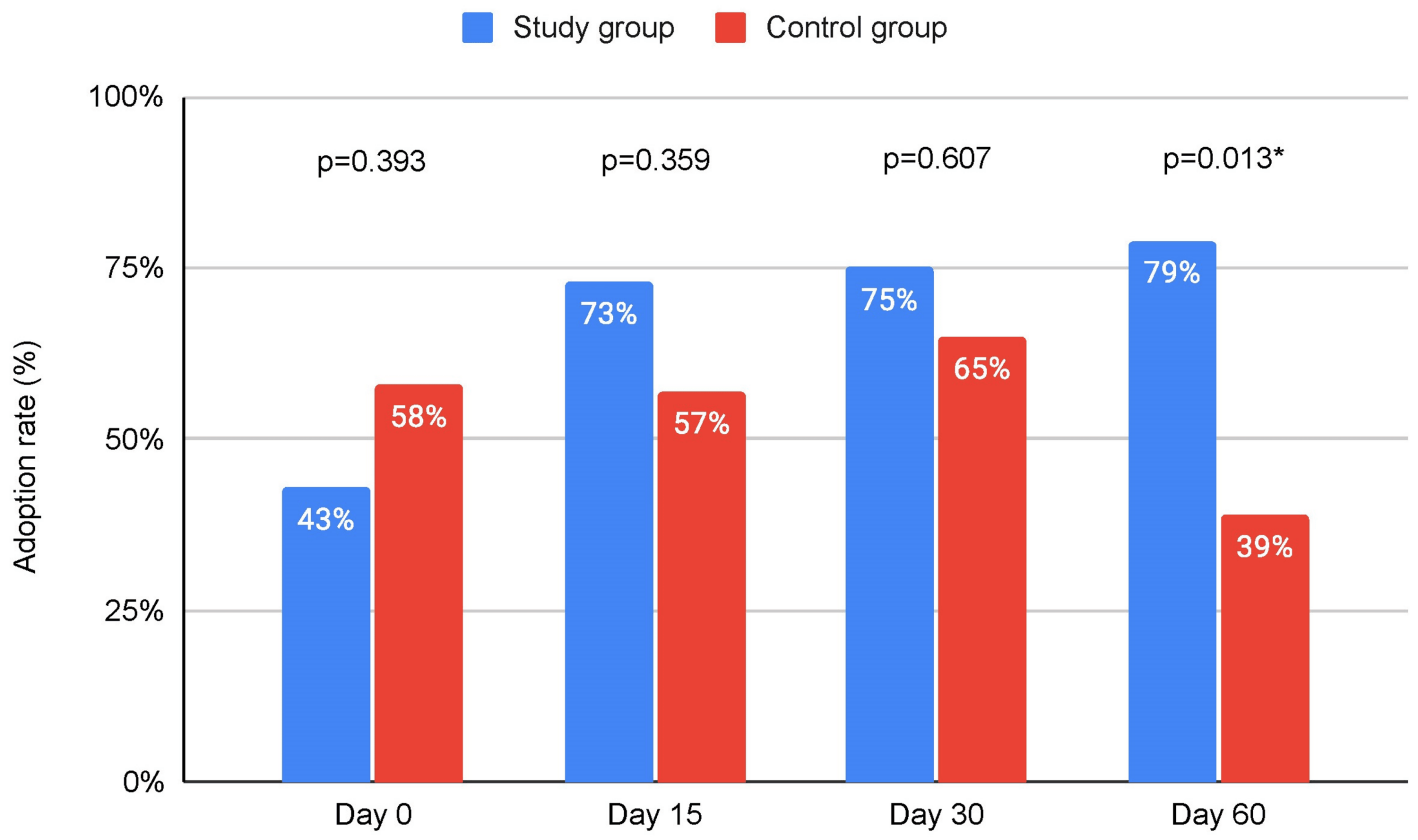
Bar chart depicting the changes in the percentage of non-exercising participants that actively pursued increased physical activity throughout their weekly routine *: statistically significant difference between study and control group (p < 0.05).

Regarding dietary habits, there was a statistically significant increase in weekly fruit portion consumption in the study group in every follow-up assessment with an upward trend as the study went on. There were no statistically significant differences with regard to weekly vegetable portions, red meat portions, or dessert portion consumption between the two groups. All observations are graphically summarized on Figure 2. Regarding exercise habits, there were no significant differences between the two groups after 15 days; however, at the 30-day assessment time point, there was a significant increase in both the number of participants that exercised weekly and the duration of the weekly exercise itself compared to the control group, with approximately twice as many participants reporting weekly exercise in the study group and on average 1.35 hours longer compared to the control group. During assessment at 60 days, the number of participants that actively exercised in the study group dropped down to similar to the control group levels, but the difference in the duration of exercise was maintained (Figure 3). All participants that reported no organized exercise were asked to report whether instead of exercise, they had at least pursued a more physically active routine. No statistically significant differences were observed between the study and control groups for the first 15 and 30 days, but the study group demonstrated a statistically significant increase in the percentage of patients that substituted organized exercise with a more physically active routine at the 60th day of assessment (Figure 4).

**Figure 5:**
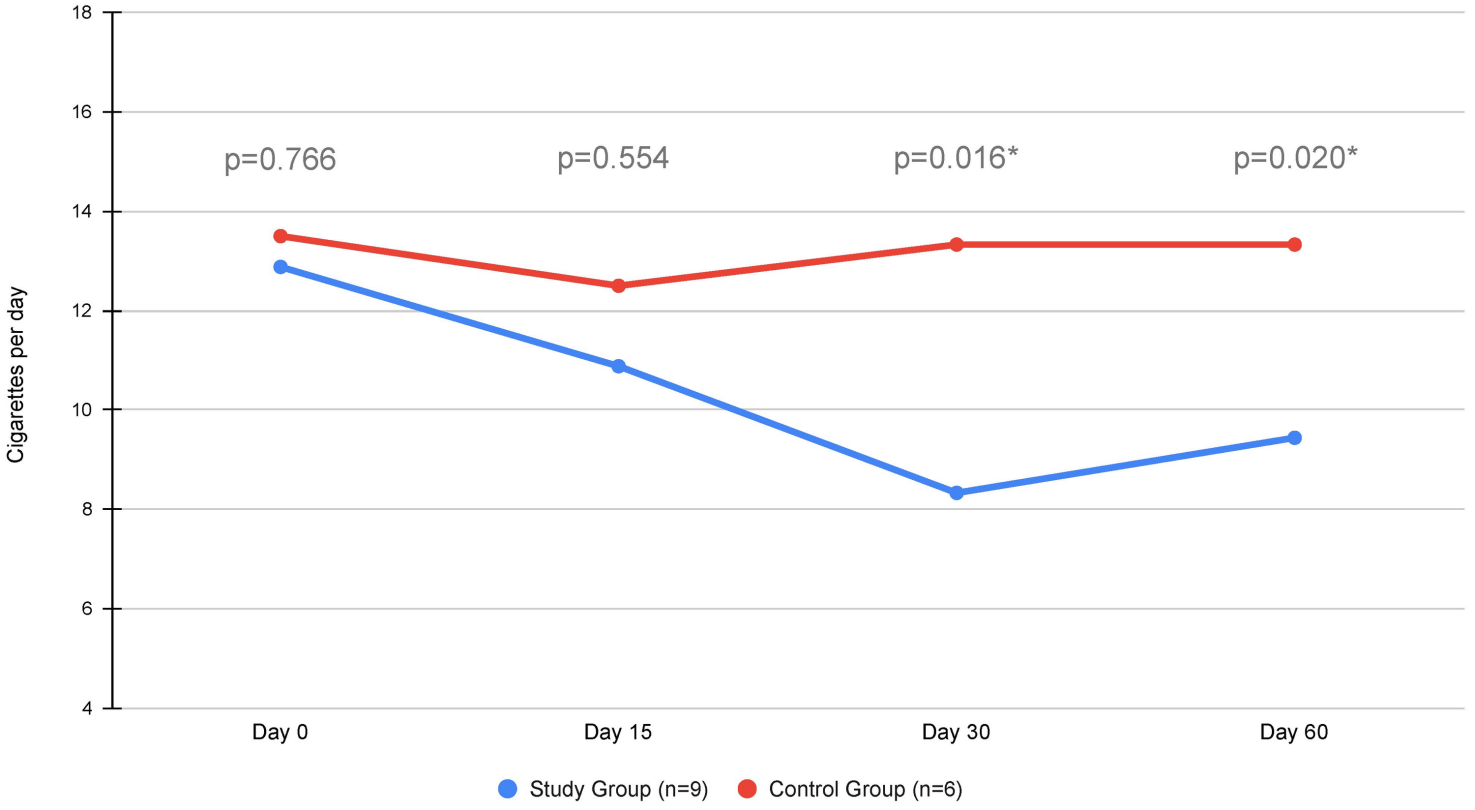
Line chart depicting the average daily number of cigarettes per smoker in the smoking subgroup of the participants. *: statistically significant difference between study and control group (p < 0.05)

A subanalysis was performed on the small number of active smokers that participated in the study and received additional lifestyle advice on cutting down and even ceasing this habit altogether. While no patients reported smoking cessation by the end of the follow-up period, smokers in the study group reported significantly fewer cigarettes per day compared to the control group during assessment at one and two months (Figure 5).

## Discussion

The present small pilot study demonstrated that lifestyle advice originating from the authority of a medical specialist has the potential to cause measurable improvements in patient quality of life and daily habits. In the present study, while all patients received an in-person consultation regarding their health status and need for lifestyle modification, only the study group received further advice and encouragement on a consistent basis for two months following their initial visit. These women, while having on average the same quality of life parameters on initial assessment, demonstrated statistically significant differences in quality of life compared to controls during the first follow-up assessment (15 days) in half of the assessed domains. This difference was maintained in 30 days, but no significant differences in overall scores were apparent after 60 days. Regarding lifestyle modification, women in the study group significantly increased consumption of weekly fruit portions, while exercise and smoking habits also improved. Overall, habits and lifestyle improvement were not as prominent as the improvement in the reported quality of life.

With regard to the observed improvement in quality of life, literature data confirm that lifestyle interventions can exert such an effect, particularly in patients suffering from metabolic disorders and/or cancer. A large meta-analysis demonstrated a small to medium positive impact of lifestyle interventions on the quality of life of such patients, with a cumulative Hedge’s g of 0.38 (95% CI 0.25-0.50; Z = 5.94; p < 0.001) [12]. The positive effect was even more pronounced in female participants with a strong positive impact; a Hedge’s g of 0.59 (95% CI 0.29-0.90; Z = 3.81; p < 0.001) [12]. While in the present study, only some of the participants had a history of metabolic disorders and/or malignancy, the overall significant improvement in perceived quality of life is definitely indicative of the potential of such interventions in the context of prevention as well. In the present study, the impact was stronger during the initial first month but was not maintained at two months of assessment. This could be attributed a perceived “controlling nature” of the interventions, as they were provided by an external source (the clinic), which has been shown to negatively affect maintenance of the new lifestyle, compared to more autonomous, self-motivated change, which could be longer-lasting [13]. In addition, the fact that the frequency of lifestyle modification was reduced, along with a larger interval between assessments, could have impacted participant motivation. The individual domains most influenced by the lifestyle modification intervention were the physical and psychological health ones, which were maintained up to the 30-day assessment, with the psychological domain lasting even up to the 60-day assessment. This is in accordance with other studies in the literature, which report significant improvement in depressive symptoms for patients receiving lifestyle interventions, even via simple brochure [14], which was similar to the advice cards used in the present study. The improvement in physical health could correspond to the increased adoption of a fruit-rich diet and more exercise-rich routine adopted by the study group compared to controls, while the perceived improvement in social life and comfort of surroundings (environment) could be partly attributed to the known positive effect of lifestyle changes on confidence, self-esteem and empowerment [15].

With regard to measurable effects, in the present study, the lifestyle modification advice was successful in increasing the number of weekly fruit portions, an increase that was consistent and also demonstrated a non-significant upward trend throughout the duration of the study. However, this was not the case with vegetables, red meat, and desserts, which remained the same. This could potentially be attributed to the fact that fruit portions can usually be consumed as independent meals and there is no need to fundamentally amend existing nutritional plans, as is the case with vegetables and red meat. Regarding exercise habits, there were significant differences in the percentage of participants that exercised weekly at 30 days' assessment, but this was not maintained at 60 days. This failure at maintaining an exercise-rich schedule could be attributed to the focus of the intervention at motivating initiation of exercise, rather than maintenance, with the latter requiring an encouragement of a transition in the patient’s mentality after the habit is adopted [16]. However, from the subgroup of patients that maintained the exercise routine, the mean duration of weekly exercise was significantly higher in the intervention group, compared to controls, indicating that the lifestyle modification advice may have been successful for the more motivated subgroup of participants. For the subgroup of patients that did not adopt exercise, the percentage of those that consciously made their lifestyle more physically active was twice as high in the intervention compared to the control group, but it only reached this difference during the final assessment at 60 days. This difference may represent the participants who did begin exercising up until the previous assessment at 30 days, but decided to stop and instead compensated by adopting a more active lifestyle, in order to maintain some form of physical activity in their routine. This could perhaps be further investigated in a larger study, where different exercise regimens of varying levels of difficulty are offered and participants encouraged to adopt them at their own pace. Finally, while few in number, smokers in the intervention group seemed to reduce the number of daily cigarettes smoked throughout the duration of the study, indicating a positive effect of the lifestyle advice. However, it is known that long-term reduction of and abstinence from smoking is quite challenging, with a single month of reduction being insufficient evidence to safely predict complete smoking cessation [17].

The vast majority of lifestyle modification interventions utilize dedicated nutritionists, exercise coaches, and strict follow-up, which are costly and unaffordable by the average gynecological patient. The findings of the present study indicate a potential utility of simpler lifestyle modification advice in improving the quality of life and daily habits, in turn reducing metabolic disorder and cancer risk for the gynecological patient with minimal cost for them and negligible financial and human resources burden for the clinic or private practitioner. This humble beginning could constitute the motivation that patients need to seek further more individualized lifestyle modifications and exact permanent positive changes in their lives. The present study is not without limitations. First, the relatively small sample size could have impacted results; however, since this was a pilot study, it was considered an acceptable concession in order to examine the feasibility of our hypotheses regarding lifestyle modification advice. A follow-up study will be designed to include more participants and examine the validity of the observed outcomes. Second, the MQLQ-E has not been externally validated for use on a Greek-speaking population, and therefore interpretation of results may be affected. However, an internal validation was performed prior to its implementation in the study. Furthermore, since results are interpreted in the context of comparison to a control group of similar Greek-speaking patients and no direct comparisons to other studies using the questionnaire are made, this effect is somewhat mitigated.

## Conclusions

Lifestyle modification doubly benefits gynecological patients, both in terms of common metabolic disorders and in gynecological malignancy risk reduction. The present pilot study demonstrated a measurable benefit with minimal cost for the clinic. A larger study with a longer follow-up duration and a larger pool of available lifestyle modification interventions will be designed in the future, in order to validate these results and explore the prospect of implementing more interactive methods to further motivate our patients.
